# Disturbances in human position sense during alterations in gravity: a parabolic flight experiment

**DOI:** 10.1007/s00221-025-07090-z

**Published:** 2025-04-25

**Authors:** Bernhard M. Weber, Michael Panzirsch, Benedikt Pleintinger, Martin Stelzer, Stella Arand, Christian Schöttler, Ralph Bayer, Annette Hagengruber, Uwe Proske

**Affiliations:** 1 German Aerospace Center, Institute of Robotics and Mechatronics, 82234 Wessling, Germany; 2 Department of Physiology, Monash University, Clayton, Vic 3800 Australia

**Keywords:** Position sense, Proprioception, Microgravity, Hypergravity, Parabolic flight

## Abstract

Under conditions of weightlessness human position sense appears to deteriorate. This was tested, employing three methods of measurement: two-arm matching, one-arm pointing and one-arm repositioning, carried out during parabolic flight. In hypergravity (1.8G), position sense errors in a matching task increased significantly from the value during horizontal flight (1G) of + 2.5° (± 3.8° SD), to + 3.5° (± 3.3°). For pointing, errors increased significantly from + 9.1° (± 4.4°) to + 11.2° (± 4.4°). In microgravity (0G), matching errors fell significantly to + 0.35° (± 3.5°), while in pointing the fall was not significant. For repositioning, there were no significant changes in errors in either hypergravity or microgravity. It is proposed that the errors in matching and pointing are a consequence of the force of gravity acting at the elbow joint to alter the position signal coming from muscle and joint receptors. For repositioning, memory of the test angle was stored centrally, to be reproduced independently of any changes in gravity.

## Introduction

The ability to accurately perceive the position of a body part without looking at it, human position sense, is one of the proprioceptive senses, “the senses within”. Proprioception includes the senses of position, movement, force, heaviness, and balance (Proske and Gandevia 2012). Arguably, the sense of position is the most important of these, since it is believed to contribute to our self-awareness (Cole 1995) and it is an important contributor to the control of reaching movements (Desmurget et al. 1995; Sarlegna and Sainsburg, 2009).

When considering methods of measuring position sense, there is currently no agreement over the preferred method of choice. This problem was recently addressed by Roach et al. (2023) who studied three commonly employed methods. These were two-arm matching, one-arm pointing and one-arm repositioning. Somewhat unexpectedly, each method produced a significantly different outcome. This led to the question, do we have more than one position sense?

We have maintained a continuing interest in position sense over the years and, more recently, become aware that under conditions of weightlessness position sense is disturbed (Young et al. 1984; Kenyon and Young 1986; Weber et al. 2020). It has led us to speculate about possible mechanisms responsible for the disturbance (Weber and Proske 2022; Proske and Weber 2023). In the light of the findings by Roach et al. (2023) we have decided to re-measure position sense during changes in gravity with each of the three methods and to look for differences in outcomes. Here we were hoping to obtain new insight into the neural mechanisms underlying the generation of position sense.

Arguably the most influential study of the effects of changes in gravity on position sense is that by Lackner and DiZio (1992). Position sense was measured, using a two-arm position matching task, during the rises and falls in gravity during parabolic flight. However, rather than studying position sense directly, the authors made observations on the illusory changes in perceived limb position in response to muscle vibration (Goodwin et al. 1972). The method has the advantage of producing large changes in position sense for which the influence of gravity can be readily studied. Other reports on position sense in microgravity were those of Bock (1994) and Bringoux et al. (2012).

The explanation preferred by Lackner & DiZio for their findings was that spindle output per unit stretch of arm muscles was influenced by G-level. Spindle rates can be altered by means of muscle length changes and by fusimotor activity. The authors proposed that gravity-dependent vestibular stimulation led to changes in fusimotor activation of spindles in forearm muscles and this altered the vibration responses. Our own hypothesis for the gravity effects was based on the findings of Bringoux et al. (2012). They showed that errors were made in a reaching task, carried out during parabolic flight, where participants overshot the target in hypergravity and undershot it in microgravity. Adding gravity-like torque, by means of elastic straps stretched across the arm before and during the movement recovered participants’ performance in microgravity to resemble that in normal gravity. The authors postulated that in microgravity the increased joint torque generated by the elastic straps enhanced arm position sense.

We have considered the possibility that the normal position signal, especially near the flexion or extension limits of a joint’s working range, was likely to include inputs from both spindles and joint receptors (Proske 2023). In hypergravity, torque levels at the joint would be expected to increase, to raise the joint receptor component of the position signal and therefore lead to increases in position sense values above those for 1G levels. Similarly, a fall in joint torque would lower the joint receptor component and therefore reduce position sense values below 1G values. With this mechanism in mind, we compared position sense values using the three methods and asked the question, were all three similarly affected by the changes in gravity?

In the present study we have chosen to measure position sense directly, rather than as a vibration illusion. In view of the findings of Roach et al. (2023), we have considered the possibility of the existence of more than one position sense (Weber and Proske 2022). It has led us to re-examine the question of the effects of weightlessness on position sense, more broadly. Participants were asked to measure position sense with each of the three methods, during periods of high and low gravity generated during a series of parabolic flights. The aim was to try to confirm, during periods of high and low gravity, the disturbance of position sense, measured with each method.

## Methods

### Sample

12 adult participants (1female, 11male) 26–53 years old (M = 37.9 years, ± 8.2 SD), took part in the present study. Participation was voluntary. One of the participants had previously taken part in similar parabolic flight campaigns. None of the participants performed regular fitness exercises with their arms or reported current or past arm injuries. All participants underwent a medical assessment before taking part in the flight campaign. In addition, their health status was checked on the first day of the week of the flight campaign by an aviation physician. Participants were informed in advance about the experimental tasks and procedures and all provided written, informed consent. The experimental protocol was approved by a French ethics committee (“Comité de protection des personnes Sud-Méditerranée I”; ID RCB number: 2023-A01115-40). Ethical aspects of the project conformed with the Declaration of Helsinki.

## Apparatus

### Experimental setup

**Fig. 1:**
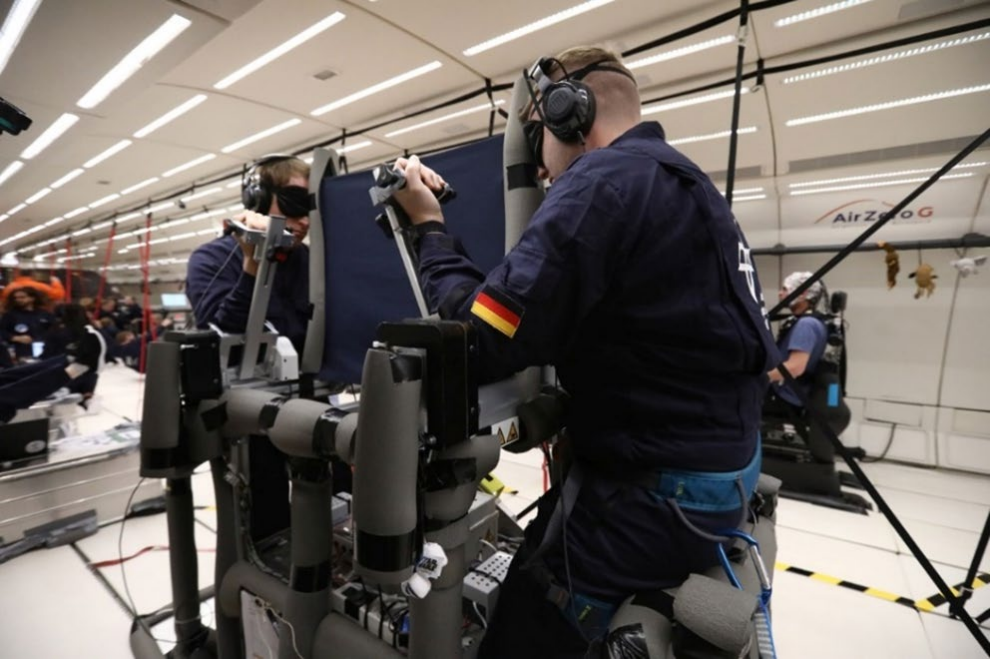
Experimental Setup: Two participants sat facing one another in the set-up. Both gave written consent to publication

The experimental setup consisted of an equipment rack fixed to a base plate and two seats also on base plates, all bolted firmly to the floor of the aircraft. In the equipment rack, all necessary hardware components (personal computer, motor controllers, electronical components etc.) were firmly locked in place. Two paddle setups, on each side of the equipment rack, were installed, facing each other, so that two participants could perform the experiment at the same time (see Fig. 1). Each setup’s height could be adjusted individually to suit the participant. In front of each participant were two paddles, which could be moved freely in the sagittal plane over a range of 90° from the horizontal (0°) to the vertical position (90°). Mechanical stops prevented movements beyond this range. Both paddles could be automatically moved to predefined angles by integrated motors. For one of the two paddles and its motor, an electromagnetic clutch was incorporated which, when released, allowed the paddle to move freely, without any frictional or inertia effects from the motor. Indicated angles were recorded by potentiometers with an angular resolution of ± 0.3°. Selected positions of both paddles could be locked in place by motor brakes. At one end of each paddle was a soft elbow support (with a Velcro strap to hold the elbow in position) and at the other end there was a padded hand rest with a handle. The elbow rest was positioned so that the elbow pivot point and the paddle’s axis of rotation were aligned. The distance between elbow rest and hand rest as well as the distance between handle and paddle could be individually adjusted.

To ensure body stabilization during microgravity, the participants’ feet were strapped to the base plate of the experimental setup. To stabilize participants during the hypergravity phases, a seat support was placed behind the participant which they could lean against or sit on (see Fig. 1). All structures were padded with foam for protection. At one side of the rack, the experimenter’s working area was set up on the floor, equipped with a laptop for monitoring and controlling the experimental procedures.

### Experimental software

**Fig. 2:**
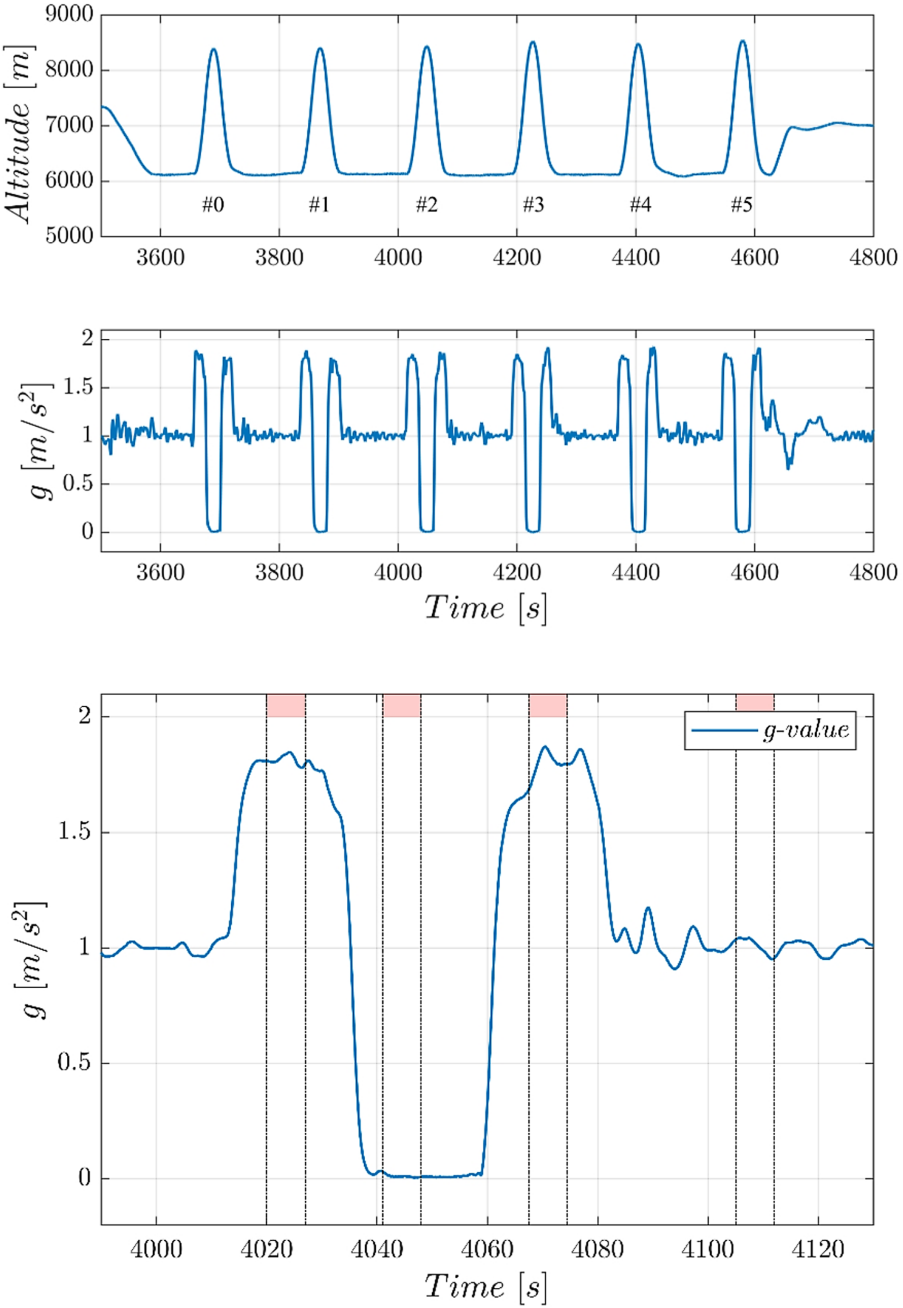
Examples of flight recordings. Top panel: parabolae timing and altitudes: Familiarization parabola #0 and first block of 5 experimental parabolae. Second panel: recorded g-values for these parabolae. Third panel: expanded view of g-values for a single parabola (parabola #2): g values in blue, red bars indicating start and end of experimental trials

The different gravitational phases during the parabolae (1.8 g, 0 g, 1.8 g, 1 g) were detected using an acceleration sensor. For the first hypergravity phase, measurements were initiated after gravity values reached above 1.5 g, the microgravity phase after they fell below 0.6 g, and the second hypergravity phase when the g-value was back above 1.3 g. Finally, normal gravity was triggered when gravity values had returned to below 1.15 g (Fig. 2). Each measurement phase was initiated only when gravity changes persisted for long enough. When conditions were suitable, the measurement proceeded, with the participant receiving their instructions via headphones. The control of the paddles as well as the evaluation of the accelerometer data was implemented in Matlab/ Simulink. The control software was executed on a real-time Linux platform at 1 kHz sampling rate. Timing of the pre-recorded instructions and paddle positions in a particular task were controlled by a JAVA program. The logging was implemented in C ++ enabling the automated identification of each parabola phase in separate files.

### The experiments

Participants completed three different experiments: (1) a two-arm matching task, (2) a one-arm a pointing task, and (3) a one-arm repositioning task. For all three experiments a test angle of 60° was used, representing approximately the middle of the forearm’s working range.

### Two-arm matching

During this task, participants were blindfolded. The experiment started with both forearms in a flexed posture (i.e. forearm at 90° to the horizontal). To begin with, forearm muscles had to be conditioned, to account for spindle thixotropic properties. With both paddles locked in position, the participant carried out isometric contractions of elbow flexors of both arms, followed by isometric contractions of elbow extensors. The contractions were approximately 20% of maximum, 0.5s in duration. To do that, participants were asked to pull both arms towards their body (“pull”), or to push them away, (“push”). When participants had relaxed, the experimental trials were started. One paddle was unlocked and the passive forearm of the reference arm was automatically moved in the direction of extension to the test angle and held in that position. Then the clutch of the other paddle opened and they were asked to bring the indicator arm into a matching position. Participants were told to move the indicator to a position where the two arms felt aligned.

### One-arm pointing

In the pointing experiment participants wore an eye patch over the eye closest to the reference arm which was also hidden behind a cloth. It meant that during pointing neither the reference arm nor its shoulder was visible to the participant. Before the reference arm was moved to the test angle, it was co-conditioned at 90°, as above. Participants carried out contractions of flexors followed by contractions of extensors and, once they had relaxed, the arm was automatically moved to a chosen test angle. The clutch of the other paddle opened and participants were asked to move this pointer paddle to a position that they felt corresponded to the position of the hidden reference arm. To do that, participants grasped the pointer paddle by its handle and moved it into position.

### One-arm repositioning

Here, participants were also blindfolded. The participant co-conditioned both antagonists of one arm with isometric contractions. Once they had relaxed, the arm was automatically moved to a chosen test angle. It was held at that angle for 2s while the participant remembered its position. Then the arm was returned to its starting position before being conditioned a second time. The clutch of the paddle was released and the participant was then asked to move their arm to the remembered position.

The pre-recorded instructions in all three conditions were given via headphones. The contractions in the 90° position were announced with “pull-push”, followed up by presentation of the test angle with the announcement, “test angle”. As soon as the clutch on the indicator side opened, the command “match” was given. Now the participant could set the desired matching angle; they had 6 s to do so and for the last three seconds there was a countdown “3-2-1”. At the end of the countdown, the angle adopted by the indicator arm was recorded. This procedure was necessary given the time limitations of the gravitational episodes.

### Experimental design

There were two experimental sessions: a pre-flight session and a flight session. During flight, participants performed experimental trials during each gravity phase of a parabola: (1) hypergravity (1.8 g), (2) microgravity (0 g), (3) hypergravity (1.8 g) and (4) horizontal flight (1 g). The pre-flight data were used to provide a baseline for comparison with results acquired during the flight.

During the flight, each participant performed the experimental tasks over fifteen parabolae. An experimental block during a parabola included each of the four gravity phases (1.8 g, 0 g, 1.8 g, 1 g). This made for a total of 60 trials over the 15 parabolae. Each of the three experiments was performed during five consecutive experimental blocks, while their order was counterbalanced across participants. The same procedure was adopted in the preflight session, except that the pauses of about 1 min 45 s between each parabola in flight were shortened to 40 s. Moreover, participants only completed two experimental blocks, i.e. two simulated parabolae for each condition; 6 blocks x 4 phases = 24 trials. The rest period between the three experiments was 100 s.

Since it is known that there are differences in proprioception between the two arms (Goble et al. 2006), participants were assigned to the two sides of the experimental rack so that 6 participants had their dominant arm as the reference, while the other 6 had their non-dominant arm as the reference.

### Procedural arrangements

All participants were informed about the background and procedure of the study and about the parabolic flights, in an online briefing one month before the experiment. 2–3 weeks before the campaign, all participants were also invited to the German Aerospace Center in Oberpfaffenhofen to receive a detailed briefing about the experimental procedure and safety instructions. In addition, the entire procedure for the three experiments was rehearsed on the apparatus.

The parabolic flights were organized and carried out by NOVESPACE at Mérignac International Airport in Bordeaux. The experimental crew and participants were briefed by NOVESPACE before flight regarding general procedures and safety regulations during the flight. Two pairs of participants took part on each of the three subsequent flight days.

On the morning of their flight day, participants completed the pre-flight session in the aircraft while on ground. During this session, participants were again reminded about the experimental task, setup and procedure. Then, the control experiment was carried out as described above.

Immediately pre-flight, all participants (experimenter and participants) received a standard dose of Scopolamine (0.7-1.0 ml for first flyers, 0.5 ml for experienced flyers). Scopolamine is the standard medication used to minimise the risk of motion sickness during parabolic flights. Side effects can be blurred vision, drowsiness, dilated pupils and dry mouth. No participants in our study exhibited counter-reactions to the drug and the subsequent experimental trials proceeded uneventfully. However, one participant experienced motion sickness during the repositioning experiment, despite having taken the drug and had to abandon the experiment. After medication, participants boarded the flight, an Airbus A310.

A complete flight took about 3.5 h and 31 parabolae were flown during this period. The first parabola (#0) was used for familiarization, i.e. participants simply lay down on the floor of the aircraft during the parabola. Then, the first pair of participants took up their positions at the setup; they were secured with a harness, positioned their feet in the foot straps and arms in the lever setup, put on the headphones and, depending on the task, the eye patch/blindfold was put in place. Meanwhile, the second pair of participants and the experimenter were sitting on the floor at the side of the rack, secured with belts to the floor of the aircraft.

The typical time sequence for each parabola was ~ 20 s hypergravity, ~ 22 s microgravity, again ~ 20 s hypergravity, and finally, back to normal gravity. The transitions between the various gravity phases were monitored by the accelerometer. When the required g-value had been reached, the experiment was started and participants were instructed via the headphones. After a break (~ 1 min 45 s), the next parabola was begun (see Fig. 2).

After each participant had completed five parabolae there was a break of 5 min, followed by a longer break of 8 min after 15 parabolae. During the longer break, the next two participants took up their position at the setup, the hand rests were adjusted and the same protocol was carried out, as before.

### Data analysis

For two-arm matching, the angles of the reference and indicator arms were recorded. The matching error was calculated as the difference between the two angles (reference angle - indicator angle), where positive values corresponded to errors in the direction of arm extension and negative values in the direction of flexion. The same was done for one-arm pointing; the angle of the hidden reference arm was compared with the pointed value. The error for one-arm repositioning was calculated as the difference between the remembered angle and the repositioned angle. For the following analyses, the recorded error measures were averaged across all trials for one experiment (i.e. 2 blocks during preflight, 5 blocks for each gravitational phase during flight).

The error measures were analysed with repeated measures ANOVA (rmANOVA) comparing the three experiments (matching vs. pointing vs. repositioning) in the preflight session and a 3 (experiment: matching vs. pointing vs. repositioning) x 3 (gravity: HG, MG, NG) rmANOVA for the flight session. Sphericity was checked using Mauchly’s test. Greenhouse-Geisser corrections were made if non-sphericity was indicated by this test. Alpha levels of post-hoc comparisons were Bonferroni-Holm corrected. In case of non-normality of the error distributions, non-parametric Friedman tests and subsequent Wilcoxon signed-rank tests with Bonferroni-Holm alpha corrections were performed. For all comparisons one-tailed tests (1tt) were performed to verify directional hypotheses, otherwise two-tailed tests (2tt) were chosen.

Data for one participant during the repositioning experiment is missing, since they had to abandon the experiment. As the result of a problem with data recording during the pre-flight session, values for two participants from one of the two experimental blocks in this session are missing.

## Results

### Pre-flight

As a first step, the data acquired on the ground (preflight session) were analysed. Since errors in the different experiments were normally distributed (Shapiro-Wilk test; *W* = 0.89), a rmANOVA could be performed, comparing the averaged position errors in the three experiments. A significant overall main effect was found in rmANOVA (*F* (2,22) = 10.99; *p* <.001; *η*^*2*^ = 0.50). Post-comparisons indicated that errors were largest for pointing (*M* = 5.95°; *SD* = 5.64°), which were significantly larger than for matching (*M* = 0.97°; *SD* = 4.0°; *p* <.0025, two-tailed testing, 2tt) and repositioning (*M* = -0.19°; *SD* = 3.96°; *p* <.007; 2tt; see Table 1).

### In-flight, single participant

**Fig. 3:**
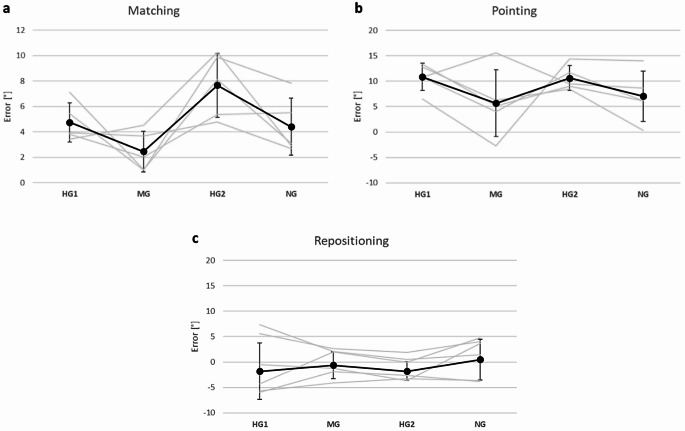
Data from a single participant. **a**: Two-arm matching, **b**: One-arm pointing, **c**: One-arm repositioning. Values for individual trials are joined as grey lines. Each line represents a single parabola. Mean error values for the five parabolae are shown as black circles (± SD). The convention was used that positive values were assigned to errors in the direction of elbow extension, negative values to errors in the direction of flexion

Examples of position sense measurements for one participant are shown in Fig. 3. During two-arm matching at 1G, (NG), the mean error for the 5 episodes of horizontal flight was 4.4°. That is, the participant matched the position of their reference arm by placing the indicator 4.4° further in the direction of extension. For the first period of hypergravity (HG 1) the mean error into extension increased to 4.8°. It then fell to 2.5° during the episode of microgravity (MG), to reach a value of 7.7° during the second period of hypergravity (HG 2). In other words, in relation to errors at 1G, there was an increase in hypergravity and a decrease in microgravity.

For one-arm pointing, the pattern of errors was similar. The NG value for the mean of the 5 trials was 7.1° in the direction of extension. This increased to 10.9° during HG 1 and to 10.6° during HG 2. In microgravity it fell to 5.7°. A feature of the errors in pointing was that while increases in errors in HG and falls in MG were smaller relative to the NG value, compared with two-arm matching, all pointing errors lay further in the direction of extension of the arm, including the value for NG. Finally, for repositioning the errors were all smaller than for matching and pointing. The NG value was 0.5°. This fell to -1.8° during HG 1, that is, the mean error was now 1.8° in the direction of flexion. The error was again − 1.8° for HG 2 and − 0.6° for MG.

### In-flight, group data

**Fig. 4:**
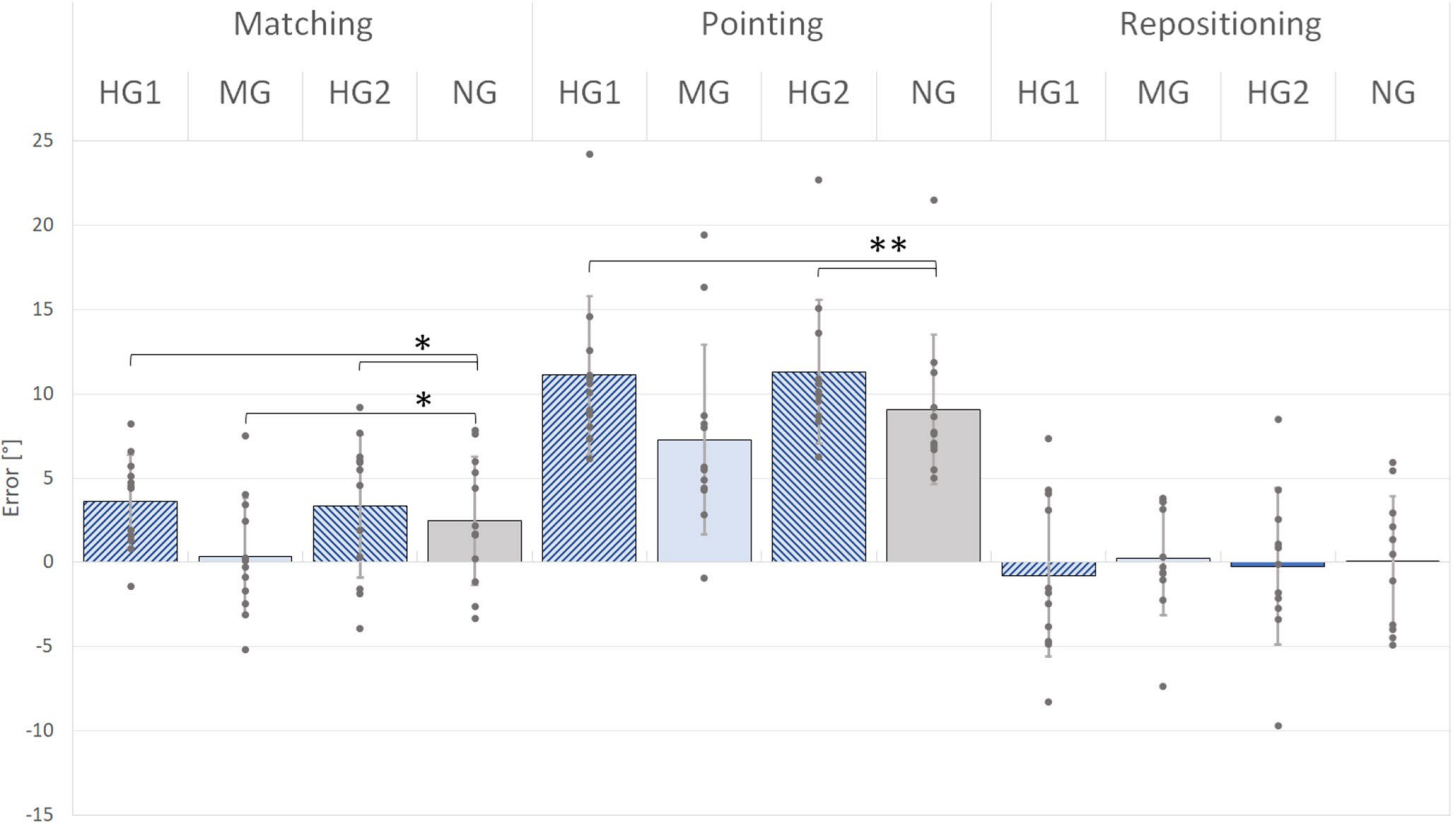
Matching, pointing and repositioning errors during the different gravity episodes for 12 participants. Display of the mean errors (± SD, shown by the error bars). Dark blue hatched columns, hypergravity (HG 1 and 2), light blue columns, microgravity (MG), grey-scale, normal gravity (NG). Values for individual participants shown as grey dots. *p <.05; **p <.01

Group errors for the 12 participants are shown in Fig. 4. It can be seen that errors in repositioning were much smaller than in matching and pointing. For pointing and matching the trend of an increase in errors during hypergravity and a decrease during microgravity, relative to the normal gravity value is clearly apparent. Values for matching were smaller than for pointing.

In-flight errors were not normally distributed (Shapiro-Wilk test; W = 0.75; *p* =.00191), making it necessary to process the data with non-parametric statistics. First, it was tested whether there was an overall effect of experiments on error values during the normal gravity episode. Friedman indicated significant differences (*χ*^*2*^(2) = 17.6; *p* <.001). Again, errors in pointing (M = 9.09°; *SD* = 4.42°) were significantly larger compared to repositioning (*M* = 0.01°; *SD* = 3.94°, *p* =.00669, 2tt) and matching (*M* = 2.48°; *SD* = 3.8°; *p* =.00665, 2tt, see Table 1) as indicated by Wilcoxon tests.

Next, the errors in the two hypergravity episodes HG1 and HG2 were compared. For all three experiments, no significant difference was found in Wilcoxon tests (*Z* = 0.39 − 0.71, 2tt). Therefore, errors in HG1 and HG2 were combined and averaged for the subsequent analysis and labelled as hypergravity condition (HG). Additionally, the preflight error values were compared to the normal gravity flight episodes (NG), but no significant difference was found when performing the Wilcoxon test in the three experiments (*Z* = 0.27–2.12; 2tt).

Further analysis used only the in-flight values for normal gravity (NG). The reason was that during flight there were small fluctuations in g-values (see Fig. 2). For this reason, and in order to take into account possible effects of Scopolamine administration, comparisons were limited to within flight.

Next, the normal gravity episodes (NG) were compared with the microgravity (MG) and hypergravity (HG) episodes, (see Table 1; Fig. 4).

For *matching*, two Wilcoxon tests were performed, indicating that error values were significantly lower (i.e. less forearm extension) for MG (*M* = 0.35°; *SD* = 3.50°) compared to NG (*M* = 2.48°; *SD* = 3.80°; *p* =.0121, one-tailed testing, 1tt). On the other hand, for HG, error values were significantly higher (*M* = 3.48°; *SD* = 3.29°; *p* =.0497, 1tt) compared to the NG baseline.

Wilcoxon comparisons for *pointing* showed a trend for error values to be lower in MG (*M* = 7.28°; *SD* = 5.63°) compared to the NG values (*M* = 9.09°; *SD* = 4.42°), although the required level of significance was not reached (*p* =.0583, 1tt). Again, error values for HG were significantly higher (*M* = 11.21°; *SD* = 4.38°) compared to NG (*p* <.00222, 1tt).

For the *repositioning task*, the two Wilcoxon tests comparing NG (*M* = 0.01°; *SD* = 3.94°) with HG (*M* = -0.50°; *SD* = 4.62°) and MG (*M* = 0.23°; *SD* = 3.35°) did not reveal any significant differences.

**Table 1:** Means and SD of errors [°] for the three experiments and gravity conditions for all 12 participants. P-values for the comparison between the in-flight normal gravity baseline and hypergravity (mean of episode 1 and 2) or microgravity in square brackets. During flight, data of one participant is missing for repositioning

*N* = 12	Matching	Pointing	Repositioning
**Pre-flight**	0.97 (4.00)	5.95 (5.64)	-0.19 (3.96)
**Flight**			
Hypergravity 1	3.63 (2.78)	11.11 (4.72)	-0.78 (4.81)
Hypergravity 2	3.33 (4.23)	11.32 (4.25)	-0.22 (4.67)
Hypergravity (Comb.)	3.48 (3.29) [*p* =.0497]	11.21 (4.38) [*p* =.00222]	-0.50 (4.62)
Microgravity	0.35 (3.50) [*p* =.0121]	7.28 (5.63) [*p* =.0583]	0.23 (3.35)
Normal Gravity	2.48 (3.80)	9.09 (4.42)	0.01 (3.94)

In summary, for matching, during hypergravity position errors increased significantly compared with 1G values, while in microgravity they fell significantly. For pointing, errors during hypergravity increased significantly, but the fall in errors during microgravity failed to reach significance. By contrast, errors during repositioning showed no significant changes during either increases or decreases in gravity. In addition, the control values in normal gravity for repositioning were significantly smaller than for pointing (*p* <.003, 2tt).

## Discussion

When we embarked on the present study, it was to ask the question, did gravity influence position sense, no matter what method of measurement was used (Weber and Proske 2022)? The present study has shown that for position sense measured by matching or pointing, changes in gravity had significant effects on the measured values. For repositioning, the data suggested that position sense values were unresponsive to changes in gravity. Given these differences in outcomes, the current observations emphasize the importance of declaring the method used whenever position sense is measured, both at ground level and under conditions of weightlessness. Our observations suggest that of the three methods used in the present study, with the method of repositioning, it is not possible to reveal any disturbance of position sense by gravity and that if gravity effects were to be studied further, the preferred methods to use would have to be matching or pointing.

In Roach et al. (2023), before each measurement, elbow muscles were conditioned in such a way that it brought out a thixotropic pattern in the distribution of the position errors. Since muscle spindles are the only known sensory receptors to exhibit thixotropy (Proske et al. 1993), such patterns were interpreted as evidence for spindles being involved in generation of the position signals. There was evidence of spindle participation in position sense measured with two-arm matching and one-arm pointing, while for repositioning the evidence was weak. Such an outcome reflected a similar pattern to that seen with gravity effects in the present study. It tempted us to say that when spindle participation in a measurement could be demonstrated, this made it likely that gravity effects could be revealed as well. It was as though in a measurement of position sense the gravity effects were linked in some way with the participation of spindles. That conclusion strengthened our view that spindles played a key role in the observed gravity-dependent changes in position sense.

In the present study, for matching and pointing there were increases in errors in hypergravity and decreases in microgravity. What might that mean? As mentioned, it is believed that muscle spindles provide the position signal during movements about a joint; an increase in spindle discharge signals a longer muscle, a more flexed or extended joint (Matthews 1988). We suggest that there is a spindle discharge - joint angle relation for the determination of position sense, established during development (Held and Bauer 1967). If, as a result of an increase in gravity, the position signal increases, this would be expressed in both arms, the reference arm sitting at 60° and the indicator moved by the participant. Since the reference remains fixed at the test angle, the signal coming from it would be higher than expected from 1G values. The indicator would move towards the position of the reference, until a point was reached where the signals from the two arms matched. For elbow flexors this would be a position where the indicator arm was more extended than the reference. If spindle discharge in elbow flexors decreases in microgravity, this would lead to a fall in position errors, producing values for elbow extension below those for 1G levels.

This argument does not consider the spindle signals in the antagonist extensors. Presumably, in real life, it is the effect of gravity on the balance of discharges in flexors and extensors which determines the direction of the errors. However, in the present study, all of the errors for matching and pointing were in the direction of elbow extension (Fig. 4), including the values in normal gravity, suggesting that the signal coming from the flexors dominated the outcome.

It could be argued that the smaller errors in microgravity represented a more accurate measurement and not be a disturbance at all. We propose that whenever the prevailing spindle discharge rates are altered, up or down, away from their normal 1G level, this should be seen as a disturbance. These gravity-dependent alterations in the spindle rate: joint angle relation lead the participant to perceive their arms in positions which are unexpected and therefore makes them unsure of the reliability of their movements when these are made in the absence of vision.

Here it should, perhaps, be remembered that there is some evidence for gravity-based influences acting on position sense measured at ground level, under 1G conditions. When participants were asked to judge elbow position with respect to the vertical, they performed better than when asked to focus on joint angles (Soechting 1982).

Why should spindle discharges increase or decrease during gravity changes? In an arm reaching task it was shown by Bringoux et al. (2012) that during parabolic flight reaching errors were made with changes in gravity. Participants overshot the target in hypergravity and undershot it in microgravity. Adding gravity-like torque, by means of elastic straps, stretched across the arm before and during the movement, recovered participants’ performance in microgravity to resemble that in normal gravity. The authors postulated that in microgravity the increased joint torque generated by the elastic straps enhanced arm position sense. One possible reason for this was an increase in skeletomotor activity required to overcome the additional torque generated by the straps and this would be accompanied by co-activated fusimotor activity that raised spindle discharges.

We have suggested an alternate explanation. The normal position signal, especially when generated near the flexion or extension limits of a joint’s working range, is likely to include inputs from both spindles and joint receptors (Proske 2023; Proske and Weber 2023). Joint receptors have an “activation angle” where they begin to generate a maintained discharge, which in animal preparations is 15°-20° short of the limit of movement at the joint. Therefore, when a position is adopted, which is getting closer to the joint limit, and the activation angle has been exceeded, there will be signal mixing from two sources, stretched spindles and activated joint receptors. The details have been spelt out in Proske (2024a). When hypergravity imposes extra torque on the joint, the activation angle will be moved further towards the middle of the movement range, increasing the opportunity for mixing, thereby raising the joint receptor component of the position signal. In microgravity, if the arm becomes weightless, there will be no standing torque on the joint and, as a consequence, joint receptor input will fall, lowering the position signal. Stretching elastic straps across the joint would increase joint torque and raise the joint receptor component of the position signal. That, in turn, would recover position sense values in microgravity to normal levels. To further test these ideas we plan, in the future, to measure position sense in microgravity with joint torque raised by means of elastics stretched across the elbow joint.

There is an interesting report by Motanova et al. (2022) describing construction of a “penguin axial loading suit” for use in microgravity conditions. The purpose of the suit was to create axial load, to help compensate for the lack of proprioceptive afferent feedback in microgravity. The suit incorporates a system of inbuilt elastic elements which are distributed according to the demands of selected antagonist muscle groups. The ideas underlying construction of such a suit support our joint receptor hypothesis.

In the present study, for pointing, the error values were significantly larger than for matching (Fig. 4). Not only were values larger during changes in gravity, but control errors at 1G were larger as well. A similar trend in the distribution of pointing errors was observed by Roach et al. (2023). It suggested that in normal gravity there was an offset, in the direction of arm extension, in the measured values of pointing errors. One possible explanation is that for pointing the proprioceptive information coming from the hidden reference arm must be converted to a visual frame of reference to guide the pointing arm. Such a conversion comes with additional errors when compared with a purely proprioceptive measurement (Darling et al. 2024). We suggest that such an offset was present in the parabolic measurements. A detailed explanation for the size and direction of the offset remains elusive.

The question arises, do frame of reference considerations also apply to two-arm matching? Here we have always assumed that both arms were in the same postural frame of reference (Velay et al. 1989). Certainly, the instructions to the participants were always to align the position of one arm with that of the other arm and no reference was made to gravity. Evidence in support of two-arm matching operating within a single frame of reference is the symmetrical distribution of thixotropic errors in both arms (Roach et al. 2023). From another point of view, two-arm matching is considered a low-level judgement, made within a single frame of reference (Heroux et al., 2022).

The findings for repositioning were rather different from those for matching and pointing. The errors were all rather small and there was no significant difference between position sense values during changes in gravity. Our original working hypothesis for repositioning had been that when a participant was asked to remember a chosen angle, the spindle discharge generated in arm muscles at that angle was stored in memory. Subsequently, when the participant was asked to reposition the arm, the spindle discharge for that angle was retrieved from memory and compared with the ongoing level of activity, as the arm moved towards the remembered angle, until the two matched.

However, such an explanation turned out to be wrong! The data of Roach et al. (2023) suggested that in repositioning ongoing spindle activity did not play a significant role. It raised the possibility that in generating the position signal the necessary information was likely derived from central sources (Proske 2024b). In support of that view, Roach et al. (2023) did an additional experiment where they introduced thixotropic disturbances after the memorizing stage and before the reproduction stage. The data showed that this did not alter repositioning errors. If spindles had been involved, it should have led to increases in position errors. While we cannot rule out participation of sensory receptors other than spindles in the repositioning process, our current preferred interpretation is that position information in repositioning was largely derived from central sources (Proske 2024b).

The observations made in the present experiments support the view that in repositioning there was no direct involvement of spindles. Despite the significant changes in gravity-dependent errors in matching and pointing, presumably mediated by changes in spindle afferent activity, for repositioning, errors in both hypergravity and microgravity remained non-significant. Furthermore, for repositioning, even the value during normal gravity was lower than for pointing. We conclude that of the three methods, repositioning was the most accurate and position sense values remained unresponsive to changes in gravity, suggesting that spindles played no role in this sense. Presumably, repositioning values were generated entirely centrally. In the future, we want to confirm this conclusion by repeating the repositioning experiment, but carry out the memorizing and repositioning stages in different gravity phases.

We suggest that the instruction to the participant, “Remember this angle”, leads them to focus their attention on the position of the arm, which immediately provides them with the precise angular information for that position. A memory is triggered, expressed in terms of angles of joints and lengths of muscles, but which, at the time of measurement, does not involve any ongoing spindle activity in arm muscles. The memory is referred to a central storage site for spatial information and kept there, ready for the instruction to reproduce the remembered position. These ideas are, of course, purely speculative and it is our intention to put them to the test in future experiments.

Looking more broadly, the present data support the view that different methods of measuring position sense involve fundamentally different underlying processes which will impact the meaning of a particular measurement. This is particularly relevant for the method of repositioning which is the preferred method used in most proprioceptive research and is widely employed in clinical settings. What we are learning is that the three different methods of measuring position sense are all likely to have different underlying mechanisms and it could be argued that there are several distinct position senses.

There appear to be two sources of influence determining human position sense. One is afferent signals of a peripheral origin providing information about muscle lengths and joint angles. The other is a central repository of recently remembered information concerning position of the body and its parts in egocentric and extrapersonal space, which can be accessed to provide accurate spatial information about limb position. The three methods of measurement used in the present study seem to show a progressive transition between these two influences; from one which relies almost entirely on peripheral afferent information, two-arm matching, to one that contains elements of both peripheral and central influences, one-arm pointing, to one which is concerned predominantly with central sources of information, repositioning.

Gravity appears to exert an influence on position sense only when there is evidence of a direct contribution from spindles, as seen in matching and pointing. For repositioning it appears that the central influences predominate. What is unexpected is the finding that the position values derived centrally are more accurate than those which involve spindles. The concept of a central repository of position information operating independently of peripheral influences is also novel. Presumably the stored information is acquired, in part, through memories of past kinesthetic activities.

To conclude, this study has raised a number of issues. Does gravity exert its influence on position sense through changes in torque levels at a joint, leading to an alteration of the joint receptor component of the position signal? If so, why is this not expressed in position sense measured by repositioning? Assuming the existence of a body schema as the central repository of spatial information, how is the communication carried out between the body periphery and central sites? Are spindles involved in this process? Given that the three methods studied here measure essentially the same thing, why are there such substantial differences in the underlying mechanisms? If we are right and repositioning operates substantially independently of peripheral sources of positional information, what is the significance of that? All of these issues will, hopefully, be addressed in future experiments.

## Data Availability

The dataset generated and analysed during the current study are available in the Zenodo repository, 10.5281/zenodo.13378127.
